# *RRM1* variants cause a mitochondrial DNA maintenance disorder via impaired de novo nucleotide synthesis

**DOI:** 10.1172/JCI145660

**Published:** 2022-07-01

**Authors:** Jonathan Shintaku, Wolfgang M. Pernice, Wafaa Eyaid, Jeevan B. GC, Zuben P. Brown, Marti Juanola-Falgarona, Javier Torres-Torronteras, Ewen W. Sommerville, Debby M.E.I. Hellebrekers, Emma L. Blakely, Alan Donaldson, Ingrid van de Laar, Cheng-Shiun Leu, Ramon Marti, Joachim Frank, Kurenai Tanji, David A. Koolen, Richard J. Rodenburg, Patrick F. Chinnery, H.J.M. Smeets, Gráinne S. Gorman, Penelope E. Bonnen, Robert W. Taylor, Michio Hirano

**Affiliations:** 1 Department of Neurology, H. Houston Merritt Neuromuscular Research Center, Columbia University Irving Medical Center, New York, New York, USA.; 2 Genetics Division, Department of Pediatrics, King Saud bin Abdulaziz University for Health Science, King Abdulaziz Medical City, Riyadh, Saudi Arabia.; 3 Department of Biochemistry and Molecular Biophysics, Columbia University, New York, New York, USA.; 4 Center for Biomedical Network Research on Rare Diseases, Instituto de Salud Carlos III, Madrid, Spain.; 5 Research Group on Neuromuscular and Mitochondrial Diseases, Vall d’Hebron Research Institute, Autonomous University of Barcelona, Barcelona, Spain.; 6 Wellcome Centre for Mitochondrial Research, Translational and Clinical Research Institute, Faculty of Medical Sciences, Newcastle University, Newcastle upon Tyne, United Kingdom.; 7 Department of Clinical Genetics, Maastricht University Medical Centre, Maastricht, Netherlands.; 8 NHS Highly Specialised Service for Rare Mitochondrial Disorders, Newcastle upon Tyne Hospitals NHS Foundation Trust, Newcastle upon Tyne, United Kingdom.; 9 Clinical Genetics Service, University of Bristol NHS Foundation Trust, Bristol, United Kingdom.; 10 Department of Clinical Genetics, Erasmus MC, University Medical Center Rotterdam, Rotterdam, Netherlands.; 11 Mailman School of Public Health and; 12 Department of Biological Sciences, Columbia University, New York, New York, USA.; 13 Department of Pathology and Cell Biology, Columbia University Irving Medical Center, New York, New York, USA.; 14 Department of Human Genetics, Donders Institute for Brain, Cognition and Behaviour, Radboud University Medical Center, Nijmegen, Netherlands.; 15 Radboud Centre for Mitochondrial Medicine, Department of Pediatrics, Amalia Children’s Hospital, Nijmegen, Netherlands.; 16 Translational Metabolic Laboratory, Department of Laboratory Medicine, Radboud University Medical Center, Nijmegen, Netherlands.; 17 MRC Mitochondrial Biology Unit and; 18 Department of Clinical Neuroscience, School of Clinical Medicine, University of Cambridge, Cambridge, United Kingdom.; 19 Department of Genetics and Cell Biology, Clinical Genomics Unit, and; 20 School for Mental Health and Neuroscience, Maastricht University, Maastricht, Netherlands.; 21 Department of Molecular and Human Genetics, Baylor College of Medicine, Houston, Texas, USA.

**Keywords:** Genetics, Genetic diseases, Mitochondria, Molecular pathology

## Abstract

Mitochondrial DNA (mtDNA) depletion/deletions syndromes (MDDS) encompass a clinically and etiologically heterogenous group of mitochondrial disorders caused by impaired mtDNA maintenance. Among the most frequent causes of MDDS are defects in nucleoside/nucleotide metabolism, which is critical for synthesis and homeostasis of the deoxynucleoside triphosphate (dNTP) substrates of mtDNA replication. A central enzyme for generating dNTPs is ribonucleotide reductase, a critical mediator of de novo nucleotide synthesis composed of catalytic RRM1 subunits in complex with RRM2 or p53R2. Here, we report 5 probands from 4 families who presented with ptosis and ophthalmoplegia as well as other clinical manifestations and multiple mtDNA deletions in muscle. We identified 3 *RRM1* loss-of-function variants, including a dominant catalytic site variant (NP_001024.1: p.N427K) and 2 homozygous recessive variants at p.R381, which has evolutionarily conserved interactions with the specificity site. Atomistic molecular dynamics simulations indicate mechanisms by which *RRM1* variants affect protein structure. Cultured primary skin fibroblasts of probands manifested mtDNA depletion under cycling conditions, indicating impaired de novo nucleotide synthesis. Fibroblasts also exhibited aberrant nucleoside diphosphate and dNTP pools and mtDNA ribonucleotide incorporation. Our data reveal that primary RRM1 deficiency and, by extension, impaired de novo nucleotide synthesis are causes of MDDS.

## Graphical abstract

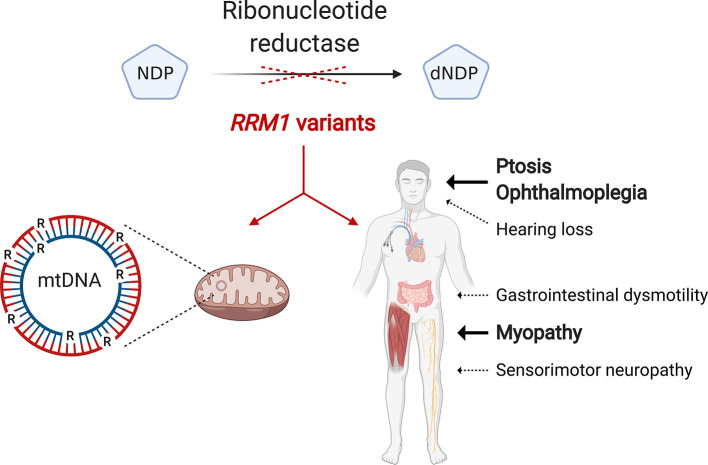

## Introduction

Autosomal disorders of mitochondrial DNA (mtDNA) maintenance are clinically heterogeneous and typically severe diseases with mtDNA depletion or multiple deletions leading to focal respiratory chain deficiency in affected tissues (1). mtDNA depletion typically causes infantile- or early childhood–onset severe multisystemic phenotypes such as Alpers syndrome, while multiple mtDNA deletions characteristically manifest later with chronic progressive external ophthalmoplegia in isolation or in combination with other organ involvement (1).

Variants in the thymidine phosphorylase (*TYMP*) gene were the first identified cause of mtDNA depletion/deletions syndromes (MDDS) linked to impaired deoxynucleoside metabolism (2). Patients with loss-of-function *TYMP* variants develop a multisystemic disorder known as mitochondrial neurogastrointestinal encephalomyopathy, which is characterized by chronic progressive external ophthalmoplegia, gastrointestinal dysmotility, cachexia, peripheral neuropathy, and leukoencephalopathy (3). Pathogenic variants in at least 6 additional nucleotide salvage pathway genes have been identified as causes of MDDS (4–9).

The salvage pathway converts DNA- and RNA-degradative intermediates into deoxynucleoside triphosphates (dNTPs) for DNA synthesis and repair. This is particularly important for mtDNA synthesis and nuclear DNA (nDNA) repair in postmitotic cells, in which de novo synthesis is downregulated (10).

Ribonucleotide reductase (RNR) catalyzes the rate-limiting step of de novo nucleotide synthesis through reduction of ADP, CDP, GDP, and UDP to their deoxynucleotide equivalents. The RNR complex is composed of catalytic RRM1 dimers in complex with RRM2 or p53R2 subunits, which fuel RRM1 with tyrosyl radicals. RRM2 expression is transiently upregulated during the S phase of the cell cycle to supply dNTPs for nDNA replication and rapidly degraded in the G_2_ phase. In contrast, p53R2 is constitutively expressed, ensuring basal de novo dNTP synthesis to complement the salvage pathway outside of S phase and in postmitotic cells (11). The functional relationship between p53R2 and the salvage pathway is notable because pathogenic variants of *RRM2B*, encoding p53R2, have been identified in MDDS (12). However, pathogenic nDNA gene variants affecting the broader scope of de novo nucleotide synthesis have not been reported.

## Results and Discussion

**Figure 1:**
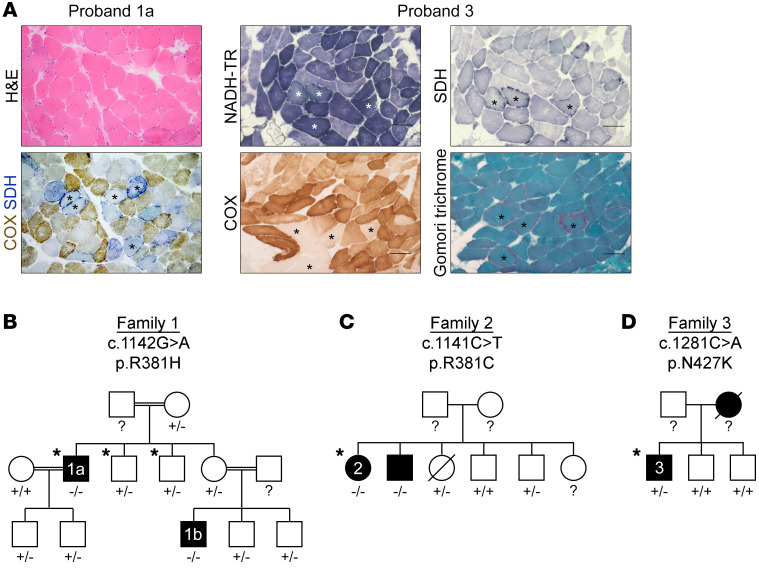
Identification of MDDS and candidate *RRM1* variants. (**A**) Histology of muscle biopsy cross sections. Asterisks indicate ragged-blue (NADH-TR and SDH), COX-negative, and ragged-red (modified Gomori trichrome) fibers. Scale bar: 100 μm. (**B**) Family 1, (**C**) family 2, and (**D**) family 3 pedigrees, indicating affected individuals (black), WES (asterisks), and Sanger sequencing (+, normal; –, variant; ?, not done).

Proband 1a, a 32-year-old Saudi Arabian man of consanguineous parents, was healthy until 7 years of age when he developed nausea and occasional vomiting (Table 1). He developed intermittent diarrhea at 14 years of age. At 17 years of age, he was evaluated for failure to gain weight and was noted to have ophthalmoparesis. Over the next 15 years, he lost 17 kg and developed moderate proximal limb weakness. Ptosis and elevated creatine kinase (697 U/L; normal 51–294 U/L) were evident after pneumonia at 31 years of age. At 32 years of age, brain MRI was normal. Absent tendon reflexes, nerve conduction studies, and electromyography indicated a mild axonal sensorimotor peripheral neuropathy. Limb MRI revealed generalized muscle atrophy. A muscle biopsy showed 50%–60% COX-deficient fibers, 3%–4% ragged-red fibers (RRFs), numerous highly atrophic fibers, regenerating central nucleated fibers, and a few fibers with necrotic sarcoplasm (Figure 1A). Southern blot revealed multiple mtDNA deletions (Supplemental Figure 1A; supplemental material available online with this article; https://doi.org/10.1172/JCI145660DS1). Proband 1b, a nephew of proband 1a, developed ptosis, ophthalmoparesis, cachexia, and intestinal dysmotility (Table 1).

Proband 2 was a healthy Dutch woman until 45 years of age when she developed double vision, ptosis, ophthalmoplegia, myopathy, and impaired gait (Table 1). She was diagnosed with MDDS following a muscle biopsy that revealed multiple mtDNA deletions and reduced mtDNA (10%–30% of age-matched controls) (Supplemental Figure 1A).

Proband 3 was a White British man; his symptoms began in his early 40s, with double vision and ptosis, which progressed to ophthalmoplegia, dysphagia, sensorineural hearing loss, and muscle atrophy (Table 1). Although creatine kinase levels were normal, muscle biopsy revealed COX-deficient fibers and RRFs (Figure 1A). Southern blot and long-range PCR of muscle DNA identified multiple mtDNA deletions (Supplemental Figure 1, A and B).

Proband 4 was a 61-year-old woman of nonconsanguineous parents with double vision, ptosis, ophthalmoplegia, and difficulty walking stairs and standing due to muscle weakness. In retrospect, she perceived weakness since childhood, though it did not interfere with daily activities. Muscle histopathology showed COX-deficient fibers and 30% RRFs. Molecular analysis revealed multiple mtDNA deletions.

To investigate the genetic cause of MDDS in proband 1a, we performed whole exome sequencing (WES) and identified a candidate *RRM1* missense variant (NM_001033.5: c.1142G>A; NP_001024.1: p.R381H). Arginine 381 was highly conserved (Supplemental Figure 1C), and in silico PROVEAN and SIFT (13, 14) predicted that the p.R381H variant was deleterious (Supplemental Figure 1D). In addition, the *RRM1* c.1142G>A single nucleotide variant was absent in gnomAD v2.1.1 (Genome Aggregation Database, https://gnomad.broadinstitute.org/). Familial segregation studies found that of 11 relatives only probands 1a and 1b were homozygous for c.1142G>A, whereas no others reported similar clinical manifestations (Figure 1B).

WES of proband 2 independently identified a homozygous *RRM1* missense variant (c.1141C>T; p.R381C) affecting the same amino acid residue identified in family 1 probands. This variant was present in gnomAD at a very low frequency (0.0001) in heterozygosity but was absent in homozygosity. Sanger sequencing revealed that her similarly affected brother was homozygous for c.1141C>T (Figure 1C). WES of proband 4 revealed an identical homozygous *RRM1* variant.

The candidate variant of proband 3 was a heterozygous missense variant (c.1281C>A; p.N427K), affecting a highly conserved amino acid residue (Supplemental Figure 1C). PROVEAN and SIFT predicted p.N427K to be deleterious (Supplemental Figure 1D). Furthermore, this variant was absent from external databases, including gnomAD. Both unaffected brothers of proband 3 did not harbor the c.1281C>A variant (Figure 1D). The deceased mother of proband 3 reportedly had similar clinical manifestations, though her genotype could not be ascertained.

**Figure 2:**
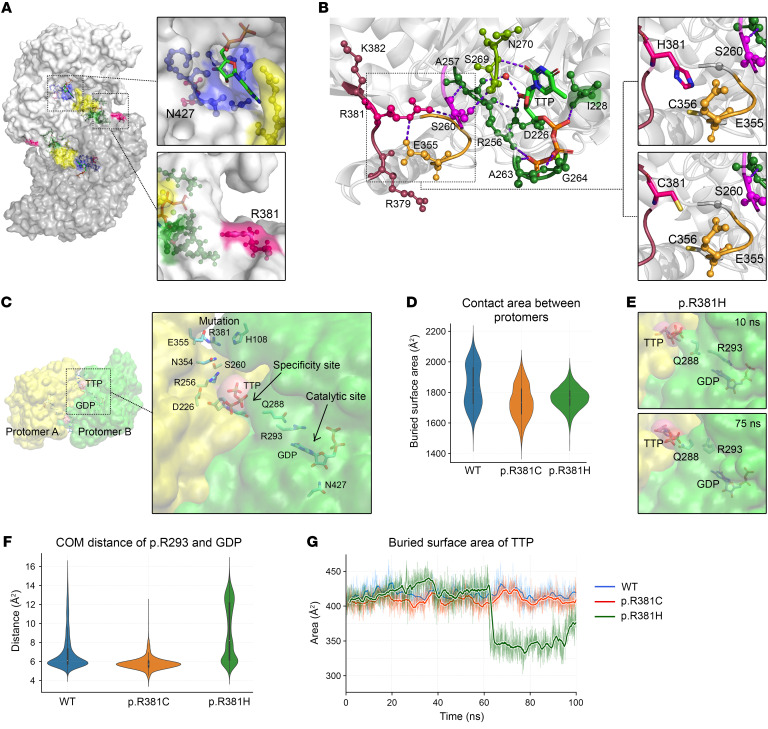
Structural analysis and MD simulations of *RRM1* variants. (**A**) Surface rendering of RRM1 dimer with highlighted catalytic site (blue), specificity site composed of loop 1 (green) and loop 2 (yellow), and loci of RRM1 variants (pink). (**B**) Hydrogen bonds between p.R381 and the specificity site via p.E355 and p.S260. (**C**) RRM1 protomers (yellow and green), ligands TTP and GDP, and allosteric communication bridging the mutation, specificity site, and catalytic site. (**D**) Interprotomer BSA of WT, p.R381C, and p.R381H, with median values of 1830.6 Å^2^, 1734.8 Å^2^, and 1764.2 Å^2^, respectively. (**E**) Snapshots of the p.R381H MD simulation capture the reorientation of p.R293 and p.Q288. (**F**) Conformational distance between the p.R293 side chain guanidinium group and GDP. (**G**) Buried surface area of TTP.

The structural basis of RRM1 function has been well-characterized (15, 16). We explored candidate variants in the human RRM1-TTP-GDP (hRRM1-TTP-GDP) complex crystal structure (Figure 2A). Substrate binding at the catalytic site normally involves p.N427, which coordinates 2′- and 3′-OH groups of the substrate’s ribose via hydrogen bonds. The p.N427K variant disrupts those hydrogen bonds, presumably reducing substrate binding and RNR activity.

In contrast, the significance of p.R381 was obscure because it does not directly interact with described functional features of RRM1. Interestingly, p.R381 interacts with p.S260, which coordinates both flanks of loop 1 via hydrogen bonds to p.A257 and p.S269 (Figure 2B). Both p.R381H and p.R381C variants lack hydrogen bonds with p.S260, suggesting loss of potential allosteric interactions between the p.R381 locus and specificity site (Figure 2B).

Although hRRM1 and *Saccharomyces cerevisiae* Rnr1p form highly conserved tertiary structures, structural alignment shows hRRM1 p.R381 absent in Rnr1p (Supplemental Figure 2A). Nevertheless, we identified coordination between the conserved locus and loop 1 through a hydrogen bond network emerging from Rnr1p p.R379. These observations suggest that hRRM1 p.R381 is an extension of loop 1 through which the specificity site may be stabilized or fine-tuned.

To develop mechanistic insights into the effects of p.R381 variants, we performed atomistic molecular dynamics (MD) simulations using the crystal structure of the hRRM1-TTP-GDP complex in its dimeric form. We modeled a disulfide bond between p.R381C and p.C356 due to their proximity (Figure 2B). All systems were stable throughout the simulations, as indicated by time evolution root mean square deviation median values below 3 Å (Supplemental Figure 2, B and C).

We focused on an extensive network of hydrogen bonds between p.R381 and the catalytic site “gating” p.R293 (17) (Figure 2C). Although p.R381C did not disrupt the hydrogen bond network, it did reduce protein flexibility, as indicated by the root mean square deviation reduction from 2.72 Å to 2.50 Å (Supplemental Figure 2C). The buried surface area (BSA) between the protomers decreased by approximately 100 Å^2^ (Figure 2D), indicating that p.R381C may impair RRM1 dimerization and thereby reduce RNR activity.

In contrast, p.R381H disrupted the hydrogen bond network. During the p.R381H MD simulation (Supplemental video), p.R293 underwent a reorientation away from the base ring of GDP (Figure 2, E and F). In addition, the hydrogen bond between p.Q288 and the p.R293 backbone was broken, and a new hydrogen bond was formed between p.Q288 and the specificity site TTP (Supplemental Figure 2D). This conformational change reduced the BSA of TTP (Figure 2G). Because BSA is correlated with affinity (18), results suggest that p.R381H reduces TTP binding affinity, which, combined with interruption of the hydrogen bond network, could severely impact specificity site function. Interestingly, p.R381H also reduced BSA between RRM1 protomers, suggesting impaired dimerization (Figure 2D).

**Figure 3:**
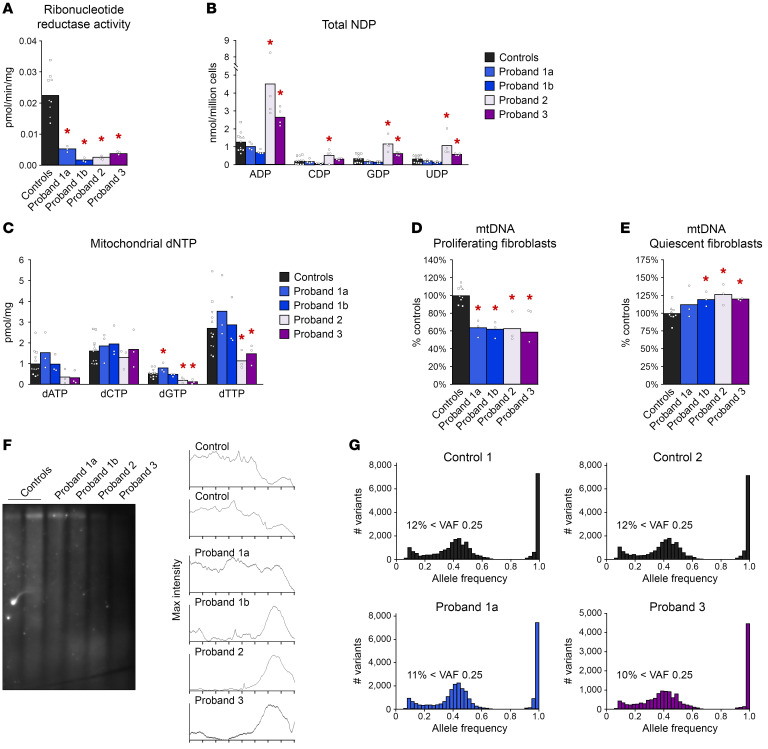
Functional characterization of *RRM1* variants in primary fibroblasts. (**A**) RNR activity of proband fibroblasts and 3 controls. Each control individual is represented by a distinct symbol. Identical symbols around each bar represent technical replicates. (**B**) Total NDPs from proliferating proband fibroblasts and 3 controls. (**C**) Mitochondrial dNTP pools of proliferating proband fibroblasts and 4 controls. (**D**) Proliferating fibroblast mtDNA quantitation. (**E**) Quiescent fibroblast mtDNA quantitation. (**F**) Southern blot indicating mtDNA ribonucleotide content. Maximum intensity plots illustrate the fragment size distribution of each lane. Representative of 3 experiments. (**G**) Variant allele frequency (VAF) histograms from WES data of proband 1a, proband 3, and 2 siblings of proband 1a as controls. We used 2-way ANOVA with the one individual per cell method to compare the mean outcome of each proband to the controls. Findings were considered as statistically significant if the corresponding *P* values were less than 0.05. *P < 0.05.

Using primary skin fibroblasts from probands, we measured RNR activity and found significantly impaired reduction of [^14^C]CDP to [^14^C]dCDP (Figure 3A). We found a similar trend of reduced RNR activity in an activity assay that we developed to increase sensitivity (Supplemental Figure 3A). By Western blot, RRM1 was similar between probands and controls (Supplemental Figure 3B).

To assess how *RRM1* variants influence nucleotide pools, we first measured total nucleoside diphosphate (NDP) concentrations (Figure 3B). Probands 2 and 3 had higher concentrations of NDPs than controls, consistent with reduced RNR activity. A similar pattern was observed for NTP concentrations (Supplemental Figure 3C). Surprisingly, fibroblasts from probands 1a and 1b had NDP concentrations similar to those of controls, and fibroblasts from proband 1b had decreased CTP and UTP (Figure 3B and Supplemental Figure 3C).

As a downstream indicator of RNR activity, we measured mitochondrial dNTPs. Fibroblasts from probands 2 and 3 had reduced dGTP and dTTP (Figure 3C). This is consistent with impaired RRM1 dimerization in proband 2 and the compromised RRM1 catalytic site of proband 3. In contrast, mitochondrial dNTP pools of fibroblasts from probands 1a and 1b were generally similar to those of controls, aside from a slight increase of dGTP for proband 1a (Figure 3C). The distinct nucleotide pools of proband 1a and 1b fibroblasts may reflect aberrant nucleotide binding at both the specificity and catalytic sites.

We then investigated the connection between RRM1 variants and mtDNA. In proliferating fibroblasts, when de novo nucleotide synthesis was most active, proband fibroblasts had low mtDNA content (Figure 3D). However, under quiescent conditions when cells rely on nucleotide salvage pathways, proband fibroblast mtDNA levels were no longer diminished (Figure 3E). To understand whether the nucleotide salvage pathway compensated for deficient de novo nucleotide synthesis in quiescent proband fibroblasts, we measured salvage pathway enzyme transcripts and found expression levels similar to controls (Supplemental Figure 4A).

Long-range PCR with proliferating proband fibroblasts did not detect multiple mtDNA deletions (Supplemental Figure 4B). Protein measurements of respiratory chain complexes showed decreased complex IV in proband 2 fibroblasts (Supplemental Figure 4C), and activities of respiratory chain complexes II+III were similarly impaired in all proband fibroblasts (Supplemental Figure 4D).

Ribonucleotide misincorporation into mtDNA is influenced by nucleotide pools and can cause mtDNA instability (19). After alkaline hydrolysis of fibroblast DNA, a denaturing Southern blot revealed smaller mtDNA fragments in probands compared with those in controls, indicating higher ribonucleotide content (Figure 3F).

Because RNR is essential for nDNA replication and repair, we assessed whether the clinical and in vitro abnormalities were primarily due to impaired maintenance of mtDNA. The absence of malignancy or defects in rapidly proliferating cells in the probands suggested nDNA replication was preserved. We measured the replication rate of proband fibroblasts and found no difference from that in controls (Supplemental Figure 5). Using WES data, we also found no differences in variant allele frequency (Figure 3G). In particular, there was no increase in the occurrence of low frequency variants, suggesting these *RRM1* variants do not increase the rate of somatic mutations.

In summary, our data reveal that both recessive and dominant *RRM1* variants contribute to the diversity of mitochondrial disorders. More broadly, our findings demonstrate that other elements of the de novo nucleotide synthesis pathway beyond RRM1 may also lead to MDDS.

## Methods

Detailed methods are provided in the Supplemental Methods.

### Study approval.

Informed consent for anonymous publication of patient clinical features and analyses of DNA samples, skin-derived fibroblasts, and muscle tissues was obtained from study participants under a Columbia University Irving Medical Center Institutional Review Board–approved protocol or with local ethics committee approval of Maastricht University Medical Centre, Maastricht, the Netherlands; University Medical Center Rotterdam, Rotterdam, the Netherlands; or University of Bristol NHS Foundation Trust, Bristol, United Kingdom.

## Author contributions

JS designed research and performed in vitro cellular and biochemical experiments. WMP, JBGC, ZPB, and JF performed structural analyses. WE, AD, EWS, ELB, PEB, KT, DAK, and RJR performed clinical experiments and diagnoses. MJF performed Sanger sequencing. JTT and RM measured ribonucleotides. CSL performed statistical analyses. PFC, GSG, RWT supervised experiments, provided clinical input, and contributed financial support. DMEIH, IVDL, and HJMS provided clinical information. MH conceptualized the study, provided clinical input, and supervised and designed experiments. JS, WMP, JBGC, ZPB, JF, and MH wrote the manuscript. All authors contributed to critical manuscript revision.

## Supplementary Material

Supplemental data

## Figures and Tables

**Table 1:**
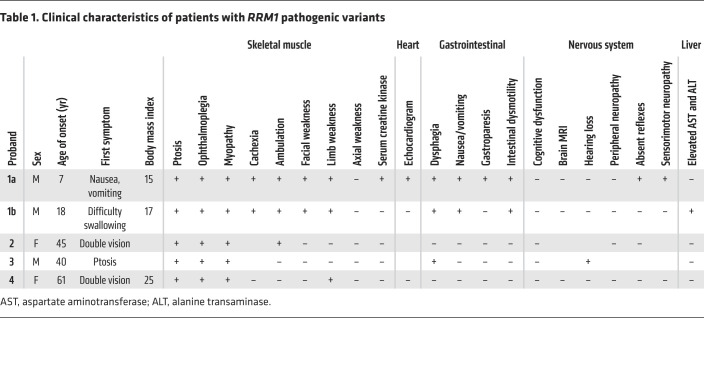
Clinical characteristics of patients with *RRM1* pathogenic variants

## Version 1 05/26/2022

In-Press Preview

## Version 2 07/01/2022

Electronic publication
